# Enhanced Generalization through Atomic Embeddings and Cross-Element Parameterization

**DOI:** 10.1021/acs.jcim.6c01725

**Published:** 2026-10-02

**Authors:** Mina Taleblou, Franco Pellegrini, Stefano de Gironcoli

**Affiliations:** 9315 Scuola Internazionale Superiore di Studi Avanzati (SISSA), 34136 Trieste, Italy

## Abstract

The limited transferability of machine-learned interatomic potentials (MLIPs) to unseen chemical compositions constrains their utility in computational materials discovery. We present a systematic investigation into two interdependent design principles—learned atomic representations and cross-element parameterization—that govern MLIP generalization. Using the diverse, multielement OC20 catalyst dataset, we evaluate chemically informed atomic embeddings against conventional 1-hot encoding within two distinct model architectures: the tensor-based LATTE descriptor and the MACE message-passing neural network. We demonstrate that embeddings such as Magpie and Matscholar reduce force mean absolute errors (MAEs) by 20–30% compared to 1-hot encoding, and improve data efficiency in low-data regimes by over 40%. Furthermore, by strategically sharing parameters across elements, we reduce the model sizes by up to 98% (from 23M to 400k parameters) without remarkably compromising accuracy, and even enable meaningful few-shot prediction for underrepresented elements. This work establishes a practical framework for building compact, transferable, and data-efficient MLIPs suitable for exploration across heterogeneous chemical spaces.

## Graphical abstract

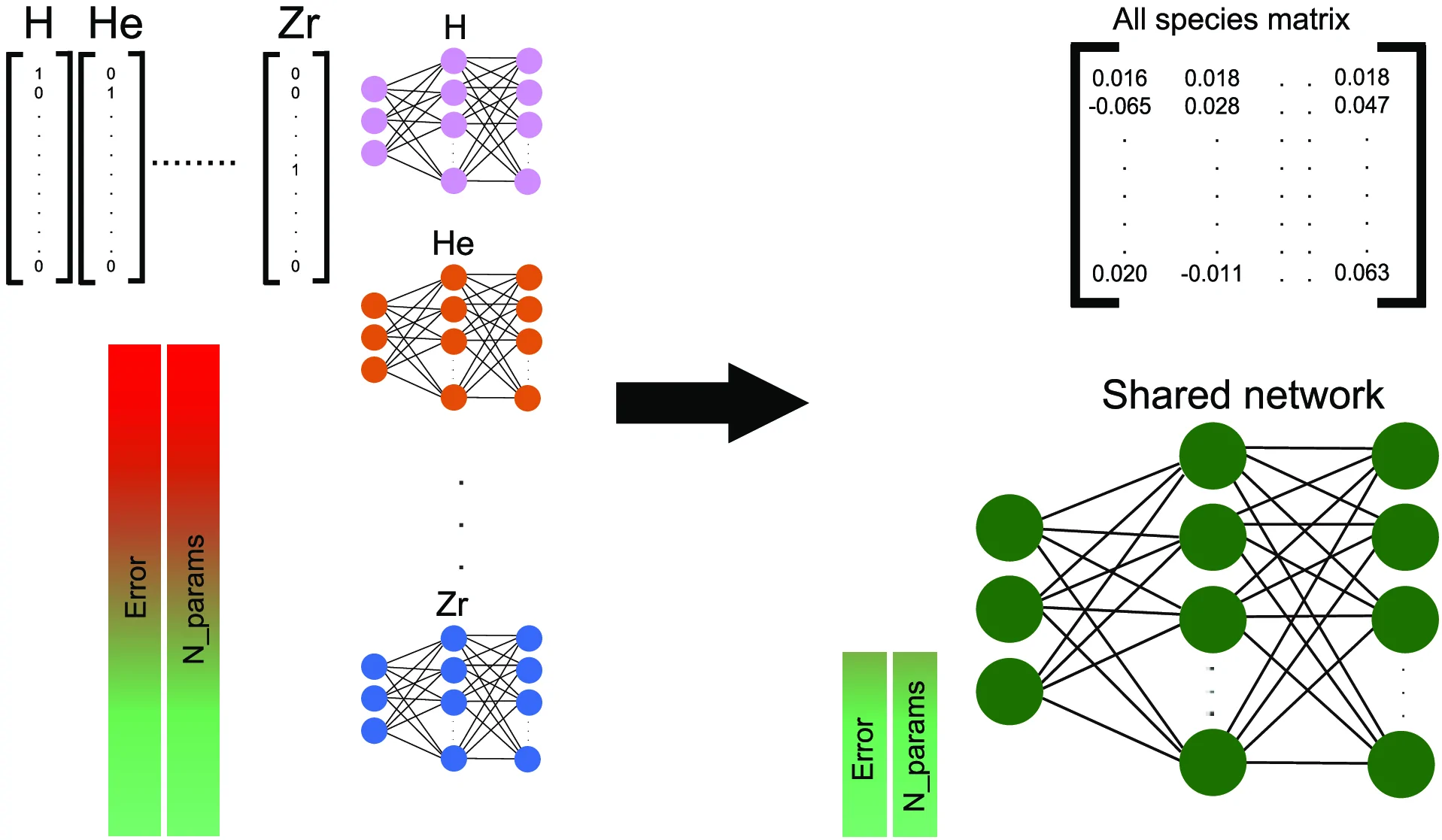

## 1 Introduction

In recent years, the application of machine learning (ML) methodologies has accelerated the discovery and exploration of materials by providing rapid, efficient, and accurate interatomic potentials.^1^ These machine-learned interatomic potentials (MLIPs) enable large-scale atomistic simulations that are computationally less demanding than first-principles methods, while capturing complex atomic interactions with high fidelity. Despite their success, a fundamental challenge persists: the ability of MLIPs to generalize beyond the chemical compositions and structural environments present in their training data—a capability critical for predictive materials design in uncharted regions of chemical space.

A key factor influencing generalization is the representation of chemical species within the model. Early approaches often employed simple encodings such as 1-hot vectors^2,3^ or atomic numbers.^4^ While sufficient for interpolation among known elements, such representations lack chemical intuition and do not capture periodic trends or similarity between elements. More recently, high-dimensional embeddings—derived from elemental properties,^5^ materials science literature,^6,7^ or unsupervised learning on structural databases^8^—have been shown to enrich model performance by providing a continuous, chemically meaningful feature space. Examples include property-based Magpie,^5^ text-based MatScholar^6^ and Mat2vec,^7^ and structure-based SkipAtom^8^ embeddings, which have improved performance in materials classification and regression tasks.^9–13^ Such representations can be categorized into physically motivated vectors, based on inherent elemental properties, and vectors more broadly learned from literature. Importantly, informative embeddings have been shown to facilitate the discovery of materials with tailored properties.^13^

Equally important is the parameterization strategy of the model. Many MLIP architectures assign separate parameters to each chemical species.^14–17^ While this can enhance accuracy for in-domain predictions, it limits transferability to unseen or rarely seen elements and increases model complexity. Parameter sharing across elements has emerged as a promising direction to improve generalization and data efficiency. For instance, in systems like *Li*_10_ *GeP*_2_ *S*_12_, shared parameters for phosphorus and germanium have been used due to chemical similarity.^18^ The SingleNN model further demonstrated that sharing hidden layers across elements can reduce training time and data requirements.^19^ However, a systematic understanding of how different levels of parameter sharing interact with various atomic embeddings remains underexplored.

In this work, we present a comprehensive investigation into the coupled roles of atomic embeddings and parameter-sharing strategies in governing the generalization performance of MLIPs. We focus on two complementary classes of models: (1) the LATTE (Local Atomic Tensors Trainable Expansion) descriptor, implemented in the PANNA package,^20,21^ which constructs atomic features via contracted tensor products and offers flexible parameterization; and (2) MACE (Message Passing Neural Networks),^22^ a state-of-the-art equivariant graph neural network. These models are evaluated on the large and diverse Open Catalyst 2020 (OC20) dataset,^23^ which contains over 1 million structural relaxations across 55 elements and includes explicit out-of-domain (OOD) splits for adsorbates, catalysts, and both—providing a rigorous testbed for generalization.

Our study addresses the following questions: *i*) How do different atomic embeddings affect model accuracy and generalization in both descriptor-based and graph-based MLIPs? *ii*) To what extent can parameter sharing reduce model size while preserving—or even enhancing—OOD performance? *iii*) Can the combination of learned embeddings and shared parameters enable few-shot or zero-shot prediction for underrepresented or unseen elements?

By answering these questions, we provide practical guidelines for developing MLIPs that are not only accurate but also transferable, data-efficient, and scalable to diverse chemical systems. The remainder of this paper is organized as follows: Section 2 describes the OC20 dataset and evaluation splits; Section 3 details the LATTE and MACE architectures, embedding methods, and parameter-sharing schemes; Section 4 presents results on embedding comparison, parameter sharing, few-shot learning, and data efficiency; Section 5 discusses the implications and limitations of our analysis, summarizes our conclusions, and recommendations for the community.

## 2 OC20: A Large, Diverse Dataset

Open Catalyst 2020 (OC20) is a large dataset generated for computational catalyst discovery applications targeting real-world challenges such as solar fuel synthesis, long-term energy storage, and renewable fertilizer production.^23^ This dataset consists of more than 1M structural relaxations and includes all the single point steps, which result in more than 260M binary and ternary catalyst and adsorbate single point calculations. This diverse dataset covers 55 elements of the periodic table, which can be a challenge for many ML models. Here, we use a subset of 2M structures out of the whole training set, and validate the trained models over four splits of data: presenting a similar distribution as in the training set or In-Domain (ID) validation set, including Out-of-Domain adsorbates (OOD-ads), OOD catalysts (OOD-cat), and OOD for both adsorbates and catalysts (OOD-both).

## 3 Models and Methods

### 3.1 The LATTE Descriptor

One of the early atomic descriptors, Atom-Centered Symmetry Functions (ACSF), was introduced by Behler and Parrinello.^14^ This descriptor consists of 2-body and 3-body terms describing local correlations around a central atom. The distance between the central atom and its neighbors is sampled by Gaussians and the angle between the central atom and pairs of its neighbors is sampled by a cosine function with flexible center and width. This descriptor, in combination with a neural network has been successfully employed for several systems with few elements.^14,24^ However, the number of 3-body terms scales quadratically with the number of atomic types and the number of neighbors. Therefore, it is almost impossible to apply this descriptor to a dataset in which there are more than a handful of species. Furthermore, information on correlation for two different chemical combinations is encoded in strictly separated places, hindering generalization opportunities. Moreover, the atomic interactions in ACSF are expandable up to 3-body; while for a reliable prediction, interactions beyond 3-body are often necessary.^25^

In this work, we employ the Local Atomic Tensors Trainable Expansion (LATTE)^20^ descriptors implemented in our code package PANNA.^21^ LATTE is inspired by Moment Tensor Potentials (MTP) proposed by Shapeev^26^ and describes the atomic environment by constructing tensors from atomic Cartesian positions and contracting them to obtain scalars. LATTE specifically provides a trainable radial function *f*_*u*,*s*_*i*__ (eq 1), which enables the user to have multiple terms based on different numbers of neighbors being involved:^20^

1 $$f_{u,s_{i}}\left(r\right)=\frac{1}{r_{u,s_{i}}^{2}}{\left[\mathrm{ReLU}\left(1-{\left(\frac{r-r_{u,s_{i}}}{w_{u,s_{i}}}\right)}^{2}\right)\right]}^{3}$$

Here, *r*_*u*,*s*_*i*__ and *w*_*u*,*s*_*i*__ are the learnable parameters that can determine the number of radial terms. ReLU (Rectified Linear Unit) yields *x* for positive values of *x*, while setting all non-positive values to zero. It is ensured that *r*_*u*,*s*_*i*__ + *w*_*u*,*s*_*i*__ < *R*_*c*_, and the function is non-zero in [*r*_*u*,*s*_*i*__ - *w*_*u*,*s*_*i*__,*r*_*u*,*s*_*i*__ + *w*_*u*,*s*_*i*__], so the contribution of neighboring atoms will be finite. The prefactor $\frac{1}{r_{u,s_{i}}^{2}}$ prevents the number of terms from growing quadratically with the number of neighbors.

Having the radial function, each tensor used to build the descriptors for the atomic environment of a given atom *i* is defined as

2 $$A_{i,u}^{\alpha_{1}...\alpha_{p}}=\sum_{j\in N\left(i\right)}\sigma_{u}\left(s_{i},s_{j}\right)f_{u,s_{i}}\left(|{\mathrm{r⃗}}_{\mathrm{ij}}|\right){\mathrm{r̂}}_{\mathrm{ij}}^{\alpha_{1}}...{\mathrm{r̂}}_{\mathrm{ij}}^{\alpha_{p}}$$

In eq 2, the index *j* runs over the atoms in its neighborhood, *N*(*i*). The chemical information regarding the central atom *s*_*i*_ and neighbor atom *s*_*j*_ is provided by *σ*_*u*_(*s*_*i*_,*s*_*j*_) = ∑_*s*_*i*_,*s*_*j*__^*u*^, a *S* × *S* learnable matrix for S species present in the dataset. *r̂*_*ij*_ is the versor of the ${\mathrm{r⃗}}_{\mathrm{ij}}$ vector from atom *i* to atom *j*. The superscripts *α*_1_...*α*_*p*_ correspond to the Cartesian components of the tensor obtained from the outer product of *p* copies of the versor *r̂*_*ij*_. *u* defines the specific instance of the tensor product corresponding to a certain set of parameters.

The product of two neighbor sums is commonly said to generate a 3-body descriptor. More generally, when all neighbor indices are distinct, the term depends on the central atom plus *K* neighbors, i.e., a (*K* + 1)-body correlation. Based on eq 3, by contracting *K* of these tensors, one obtains a (*K* + 1)-body scalar term:

3 $$B_{i,u}=\sum_{\alpha_{1}\;,\cdot \cdot \cdot ,\alpha_{m}\;}\prod_{k=1}^{K}A_{i,u_{k}}^{\alpha_{1}^{k}...\alpha_{p_{k}}^{k}}$$

Where each of the local indices α_1_, . . . , α_*m*_ is found twice among the α_*j*_^*k*^, meaning that each component of a tensor is contracted with a single other component, converting the product into a rotationally invariant scalar. The indices *u*_*k*_ imply that each term *A*_*i*,*u*_*k*__ contributing to the bin *B*_*i*,*u*_ has its own learnable parameters. The model scales linearly with the number of elements and supports arbitrary n-body interactions through the tensor ranking.

In addition to the flexibility of embedding arbitrary vectors as elemental fingerprints, LATTE offers the possibility of parameterization control at both the level of the descriptor and the level of individual layers of the neural network. In the following sections, we explore this option in detail and investigate the generalization power of the model as a function of parameterization mode.

The full details regarding the descriptor and network parameters are presented in the Supporting Information, together with the results of the different training set-ups explored in Table S1.

### 3.2 Atomic Embeddings

The description of the chemical environment of a given atom is determined by the identity and position of the surrounding atoms. In the LATTE descriptor, each term in the expansion contains trainable parameters, in particular the σ­(*s*_*i*_,*s*_*j*_) parameters in eq 2 which determine the model expressivity and adaptability to the training data. In the vanilla LATTE implementation, the weights are set independently for each atomic type (1-hot encoding). Alternatively, each atomic type can be represented by a fixed embedding feature vector in high dimension whose projection on vectors of trainable weights enters the descriptor definition.

We compare several high-dimensional atomic embeddings reported in the literature: 1-hot encoding (baseline); Random vectors (200-dimensional, sampled from a standard normal distribution); SkipAtom (200-dimensional, structure-based graph pooling);^27^ Magpie (22-dimensional vector of elemental properties);^5^ Matscholar (200-dimensional literature word embedding);^6^ Mat2vec (200-dimensional literature word embedding).^7^

### 3.3 Parameter-Sharing Strategies

The parameterization of a local environment descriptor can be taken to be different (specific) for each central atom type or can be chosen to be the same (common) for all central atoms; or its dependence on the central atom identity can be parametrized through the same embedding projection used for the surrounding atoms.

In the atomic environment formulation in eq 2, the parameterization strategy enters in the *σ*(*s*_*i*_,*s*_*j*_) term. At the descriptor-level parameterization, Table 1 shows the possible parameterization in the *σ* term of the descriptor *d*(*s*) for central *i* and neighbor atoms *j*.

**Table 1:** Modes of Descriptor Parameterization

mode	σ(*s*_*i*_, *s*_*j*_)	*d*(*s*_*i*_)	*d*(*s*_*j*_)
1h	*W* ^*s*_*i*_ *s*_*j*_^	s	s
common	W_*j*_^*s*^	c	s
emb	∑_*k*_ *W* ^*s*_*i*_ *k*^ *e* ^*s*_*j*_ *k*^	s	e
common-emb	∑_*k*_ *W* ^*k*^ *e* ^*s*_*j*_ *k*^	c	e
common-emb-central	∑_*k*_ *W* ^*k*^ *e* ^*s*_*i*_ *k*^ ∑_*k*′_ *W*′^*k*′^ *e* ^*s*_*i*_ *k*′^	e	e

In Table 1, *W* ^*s*_*i*_,*s*_*j*_^ in the first row corresponds to the fact that in the 1-hot representation, independent weights are assigned to each (*i*, *j*) species pair. In the *common* mode, the weights are specific to the environmental atoms but not to the central atom identity. In the embedding mode (*emb*), the central atom and its neighbors are represented as *1h* and embedding vectors, respectively. When parameters are commonly shared using embedding (*common-emb*), the surrounding atoms are described by an embedding vector, and there is no dependence on the central atom identity. The last mode is *common-emb-central*, in which not only neighboring atoms, but also the central atom are described by an embedding vector.

At the network-level parameterization, for a given chosen description of the local environment, the way this representation is processed can still be a matter of analysis, depending on the nature of the atom they want to describe.

When combining the atomic descriptor (*d*) and neural network with hidden layers­(*l*), we investigate several levels of parameter sharing between elements in the level of descriptor and network. For a selected network with 2 hidden layers (*l*_1_ *l*_2_) and an output layer (*l*_3_), we investigate different combinations of “*d*:*l*_1_ *l*_2_ *l*_3_”, in which parameters assignment can be specific (*s*), common (*c*), or embedding (*e*) at each level:

1h:sss: Species-specific parameters throughout.

common-emb:sss: Shared descriptor parameters, species-specific network weights.

common-emb:css: Shared descriptor and first hidden layer, species-specific network weights in the last two layers.

common-emb:ccs: Shared descriptor and both hidden layers, species-specific output layer.

common-emb-central:ccc: Fully shared parameters, with atomic embeddings used for both central and neighboring atoms.

### 3.4 Training and Evaluation

All models were trained under identical conditions on the OC20-2M dataset, with early stopping to control overfitting. Performance was evaluated using Mean Absolute Error (MAE) of energies (meV/atom) and forces (meV/Å) across the four validation splits provided in OC20: ID, OOD-ads, OOD-cat, and OOD-both.

### 3.5 The MACE Model

To verify that our findings are architecture-agnostic, we integrated embeddings into the MACE, an equivariant message-passing graph neural network.^22^

While MACE intrinsically avoids explicit *O*(*S* ^2^) species tensor products during neighbor message passing, standard implementations still rely on a learnable matrix *W*_*z*_*i*__ directly depending on the chemical element index *z*_*i*_. In our modified initialization, we generate these element-specific attributes by multiplying a continuous pre-trained elemental embedding matrix *E* and a shared learnable weight matrix *W*. While an expressive graph neural network like MACE can learn effective species representations from scratch when provided with abundant training data, parameterizing species dependence via continuous embeddings *E* provides a crucial physical inductive bias in data-scarce regimes.

This modification can be expressed as a simple matrix contraction as shown by eq 4:

4 $$h_{i}^{\left(0\right)}=A_{i}\mathrm{EW}$$

where *h*_*i*_^(0)^ is the new initial node feature, *A*_*i*_ is the original attribute of atom *i*,*E* is the chosen high-dimensional embedding matrix (e.g., Matscholar), and *W* is a learnable projection matrix. Crucially, by tuning the dimensions of the projection matrix *W*, we can convert a large, high-dimensional embedding matrix into an arbitrarily small embedding *h*_*i*_^(0)^, leading to a much smaller number of relevant learnable parameters in the model, actively enhancing data efficiency. More details regarding the hyperparameters of the MCE model are specified in the Supporting Information.

## 4 Results

### 4.1 Atomic Embedding Improves Generalization

Table 2 compares the LATTE performance when different embedding matrices are considered. A 200-dimensional random vector sampled from a standard normal distribution was included in the table as a dummy representation. This random representation yields the highest Mean Absolute Error (MAE), confirming the need for chemically meaningful representations. 1-hot encoding performs well on ID data but poorly on OOD splits. Interestingly, SkipAtom exhibits a similar limitation in generalizing to out-of-distribution structures. In contrast, Magpie and Matscholar achieve the best OOD performance, with Matscholar reducing OOD-both force errors by 22% compared to 1-hot encoding. Consequently, these two atomic embeddings are considered in the rest of this study.

**Table 2:** MAE for Energy (E, meV/atom) and Forces (F, meV/Å) Across OC20 Validation Splits

	ID		OOD-ads		OOD-cat		OOD-both	
atomic embedding/embedding size	E	F	E	F	E	F	E	F
Random/200	143	182	144	182	146	189	153	204
1-hot/200	19	83	20	86	50	131	52	142
SkipAtom/200	25	89	26	101	49	135	51	156
Mat2vec/200	27	95	29	98	44	133	45	144
**Matscholar**/200	16	79	18	81	26	103	27	111
**Magpie**/22	19	87	20	91	33	111	33	121

We also report the mean absolute error (MAE) of the total energy per structure to facilitate comparison with the original OC20 study. The total-energy MAEs for the ID, OOD-Ads, OOD-Cat, and OOD-Both validation sets are 2.135, 2.137, 2.592, and 3.077 eV, respectively. While the reported MAE gives a rough estimate of the obtained accuracy, it should be interpreted with caution, as it characterizes only the average prediction error and may obscure structure-dependent failures or rare but significant outliers. Moreover, the stability of the molecular dynamics simulations depends on the smoothness of the predicted potential-energy surface, which is not described by metrics such as MAE. Consequently, models with similar energy and force MAEs may exhibit different performance when applied to atomistic simulations. Complementary analyses, such as error distributions and uncertainty estimates, can provide a more comprehensive assessment of model performance.

### 4.2 Parameter Sharing: Reduces Model Size Without Compromising OOD Performance

A key challenge in designing transferable MLIPs is managing model complexity while preserving predictive accuracy, especially for out-of-domain (OOD) compositions. Previous studies have shown that sharing parameters during training can be beneficial,^28–30^ although determining which parameters should be shared among different elements remains a significant challenge.^31^ We investigate here how strategic parameter sharing across elements can reduce model size without degrading—and potentially improving—generalization performance.

We first evaluate the effect of parameter sharing in models using 1-hot encoding, summarized in Table 3. When descriptor parameters are shared across all elements while network weights remain element-specific (common:sss vs. 1-hot:sss), the parameter count is reduced from 23M to 16M with no loss in accuracy. More aggressive sharing, where only the output layer retains element-specific parameters (common:ccs), further reduces the model to just 400k parameters—a 98% reduction—while slightly improving OOD performance, despite a minor trade-off on in-domain (ID) data.

**Table 3:** Levels of Parameter Sharing in the 1-Hot Vector Embedded Model^a^

			ID		OOD-ads		OOD-cat		OOD-both	
*d*	*l*_1_ *l*_2_ *l*_3_	*n*	E	F	E	F	E	F	E	F
1h	sss	23M	19	83	20	86	50	131	52	142
common	sss	16M	29	83	31	86	44	123	48	134
common	css	3M	33	94	35	97	43	121	47	131
common	ccs	400k	19	93	21	96	27	115	28	125

a The corresponding models were validated on all four domains of the OC20 validation dataset. The table reports the number of parameters (n), along with the prediction errors for energy (meV/atom) and forces (meV/Å).

These results demonstrate that extensive parameter sharing is not only feasible but beneficial for generalization. The common-emb:ccs configuration achieves comparable or better OOD force and energy errors with two orders of magnitude fewer parameters, indicating that shared representations capture transferable chemical patterns across elements.

We observe similar trends when parameter sharing is applied to models using learned atomic embeddings (Table 4). For both Matscholar and Magpie embeddings, sharing parameters across descriptor and hidden layers (e.g., common-emb:ccs and common-emb-central:ccc configurations) reduces model size dramatically—to as few as 40k parameters in the fully shared Magpie model (common-emb-central:ccc)—while maintaining competitive OOD accuracy. This shows that informative embeddings complement parameter sharing, enabling compact models that retain strong extrapolation capabilities.

**Table 4:** Enhancing the Extrapolation Power of Embeddings by Sharing the Model’s Parameters

			ID		OOD-ads		OOD-cat		OOD-both	
Emb	*d*:*l*_1_ *l*_2_ *l*_3_	*n*	E	F	E	F	E	F	E	F
matscholar	emb:sss	40M	16	79	18	81	26	103	27	111
	common-emb:sss	16.5M	16	78	17	81	26	102	26	111
	common-emb:css	3M	18	86	19	89	24	102	25	111
	common-emb-central:ccc	1M	31	109	31	113	36	124	25	110
magpie	emb:sss	19M	19	87	20	91	33	111	33	121
	common-emb:sss	16M	19	86	20	89	32	110	31	119
	common-emb:css	3M	20	87	21	90	29	112	29	121
	common-emb-central:ccc	40k	34	111	34	114	41	127	30	120

Taken together, these findings underscore that parameter sharing, particularly when coupled with chemically meaningful embeddings, offers a powerful pathway to building lightweight, data-efficient, and highly transferable interatomic potentials. Similar embedding ideas were applied to mitigate the unfavorable scaling of the number of parameters in the model with the number of elements present in the dataset; however, the expression of the model was limited to 3-body interactions.^32^

### 4.3 Embeddings Enable Few-Shot Learning

We note that in the OC20 dataset, although the composition of catalysts and the combinations of catalyst/adsorbate in the validation set differ from those in the training set, all atomic species present in the validation set also appear in the training set. This scenario is common in most studies where the training and test sets are drawn from the same chemical domain, with all atomic species in the test set also present in the training set. A more strict evaluation of a model’s generalization, however, involves testing it on an element that was rarely encountered during training. While most models are carefully designed and optimized for interpolation, their poor extrapolative performance on out-of-distribution (OOD) data remains a well-known limitation. One promising strategy to address this issue involves learning element representations from large and diverse datasets, and subsequently transferring this knowledge to improve performance on OOD systems, even when only limited data are available.^3^ This concept has been applied to the simulation of inorganic reactions within a reaction graph framework, where Magpie and Matscholar embeddings were employed to represent both the precursor compounds and the constituent elements of the reaction products.^33^ Furthermore, the use of a single network architecture shared across all atomic species, combined with physically meaningful elemental embeddings, has been shown to enhance the model’s predictions for previously unseen elements.^34^

To rigorously evaluate extrapolation to rarely seen or absent elements, we designed a few-shot learning experiment centered on iron (Fe). All Fe-containing structures (≈52,000) were removed from the 2M training set, and models were subsequently retrained while gradually reintroducing Fe data in increments of 0% (zero-shot), 1, 5, 25, 50, and 100% of the original Fe subset. Performance was evaluated exclusively on a held-out validation set containing Fe structures, as shown in Figure 1.

**Figure 1:**
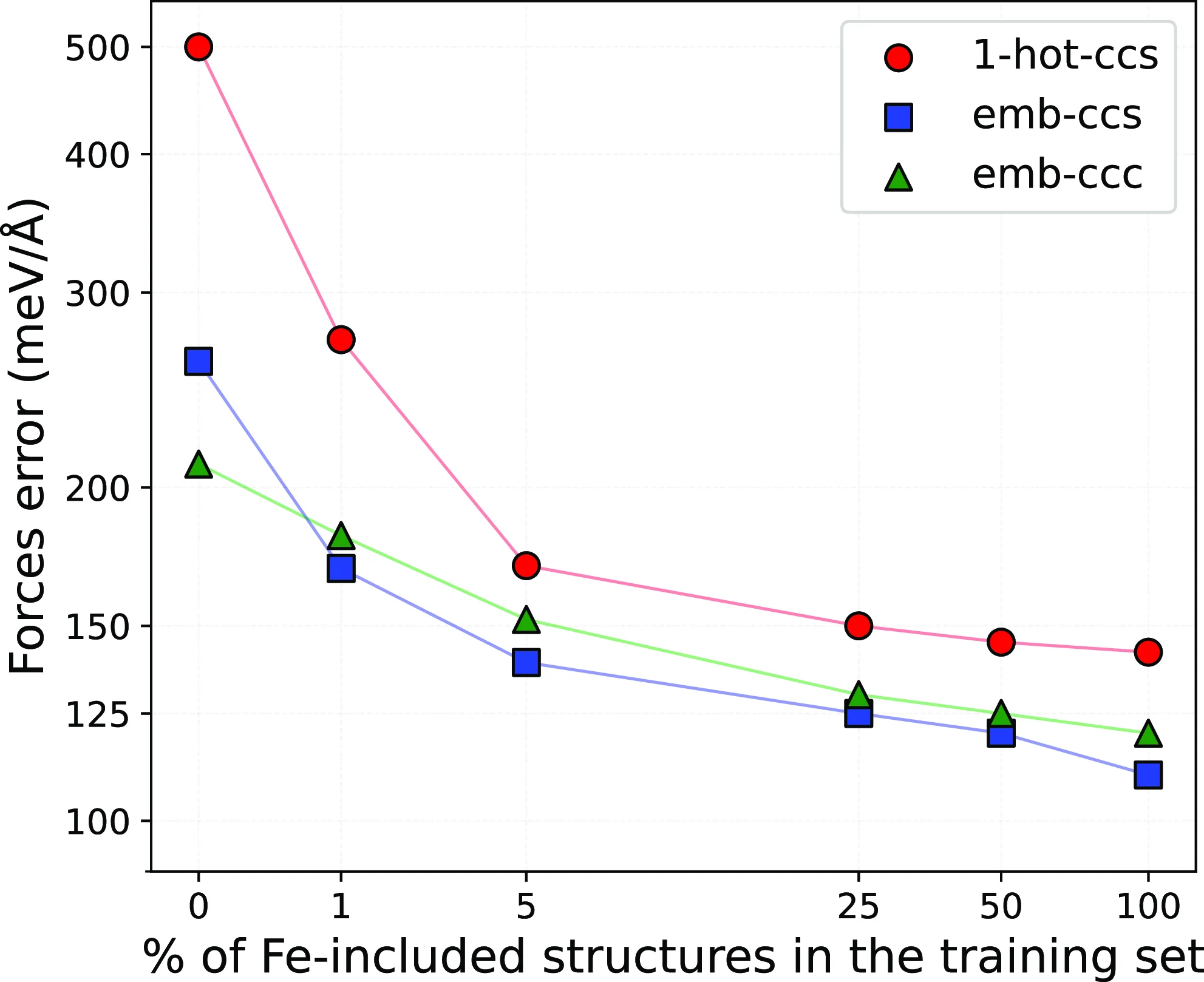
Force prediction error of 1-hot and embedded models as a function of the percentage of Fe-containing structures in the training data. The parameterization is as follows: red circles for a 1-hot embedded descriptor and sharing the parameters of the hidden layers, blue squares for the embedded descriptor and sharing the parameters in the descriptor and two hidden layers, and green triangles for sharing all parameters at the descriptor and network level while using embedding for the central atom.

Models employing element-specific parameters (e.g., 1-hot:sss) fail entirely in this setting, as the weights corresponding to the absent element remain untrained. Therefore, we focus on shared-parameter configurations that allow knowledge transfer from seen to unseen elements. For 1-hot encoding, the common:ccs scheme was used, since full parameter sharing (common:ccc) is ineffective without a meaningful continuous representation of the central atom. In contrast, Matscholar embeddings enable the fully shared common-emb-central:ccc configuration, where the central atom’s identity is encoded via its embedding vector.

As shown in Figure 1, models with shared parameters and Matscholar embeddings achieve stable force predictions with as little as 1% of the Fe training data. In comparison, the 1-hot encoded model shows higher errors and less consistent improvement as Fe data are added. This demonstrates that chemically informed embeddings provide a framework for generalizing to underrepresented elements, even when training data are extremely sparse.

For “emb-ccs” and “1h-ccs” models, the parameters in the final layer of the network are specifically assigned to each central and neighboring element. Consequently, if a particular element is absent from the training set, the corresponding parameters remain untrained, making predictions for that element unreliable. The force error reported at 0% Fe concentration corresponds to the prediction error on a test set containing structures with Fe atoms. Although the predictions for Fe atoms are effectively random due to the lack of training data, the reported MAE is averaged over the errors of both Fe atoms and the other elements present in the structures, which were included in the training set.

Although “emb-ccs” and “1-hot:ccs” configurations yield a finite prediction error for the unseen element, it is likely that the associated uncertainty is relatively high since the parameters specified for the new element are not optimized during training.

In general, combining learned atomic embeddings with cross-element parameter sharing facilitates meaningful few-shot inference for elements poorly represented in the training set. This approach moves MLIPs closer to practical zero- or few-shot capability in exploring uncharted chemical spaces.

### 4.4 Informative Embeddings enhance Data Efficiency

Appropriate atomic representations become increasingly critical when training data are limited. To evaluate the data efficiency of embedding-enhanced models, we trained LATTE on progressively smaller random subsets of the OC20 dataset­(2M, 1M, 200k, 20k, and 2k configurations). For MACE, we capped the training set size at 200k. All models were evaluated on the same OOD test set.

As shown in Figure 2 (top), for the LATTE model, the embedding-based representation consistently outperforms 1-hot encoding across all dataset sizes. With only 2k training samples, the Matscholar embedding reduced the force MAE by 19% and the energy MAE by 35% compared to 1-hot encoding, demonstrating that chemically informed embeddings dramatically lower the data requirements for achieving accurate predictions. The performance gain of embedding-based representation over 1-hot encoding is maintained as the size of the training data grows, reaching 22 and 48%, respectively, for force and energy MAE for 2M configurations.

**Figure 2:**
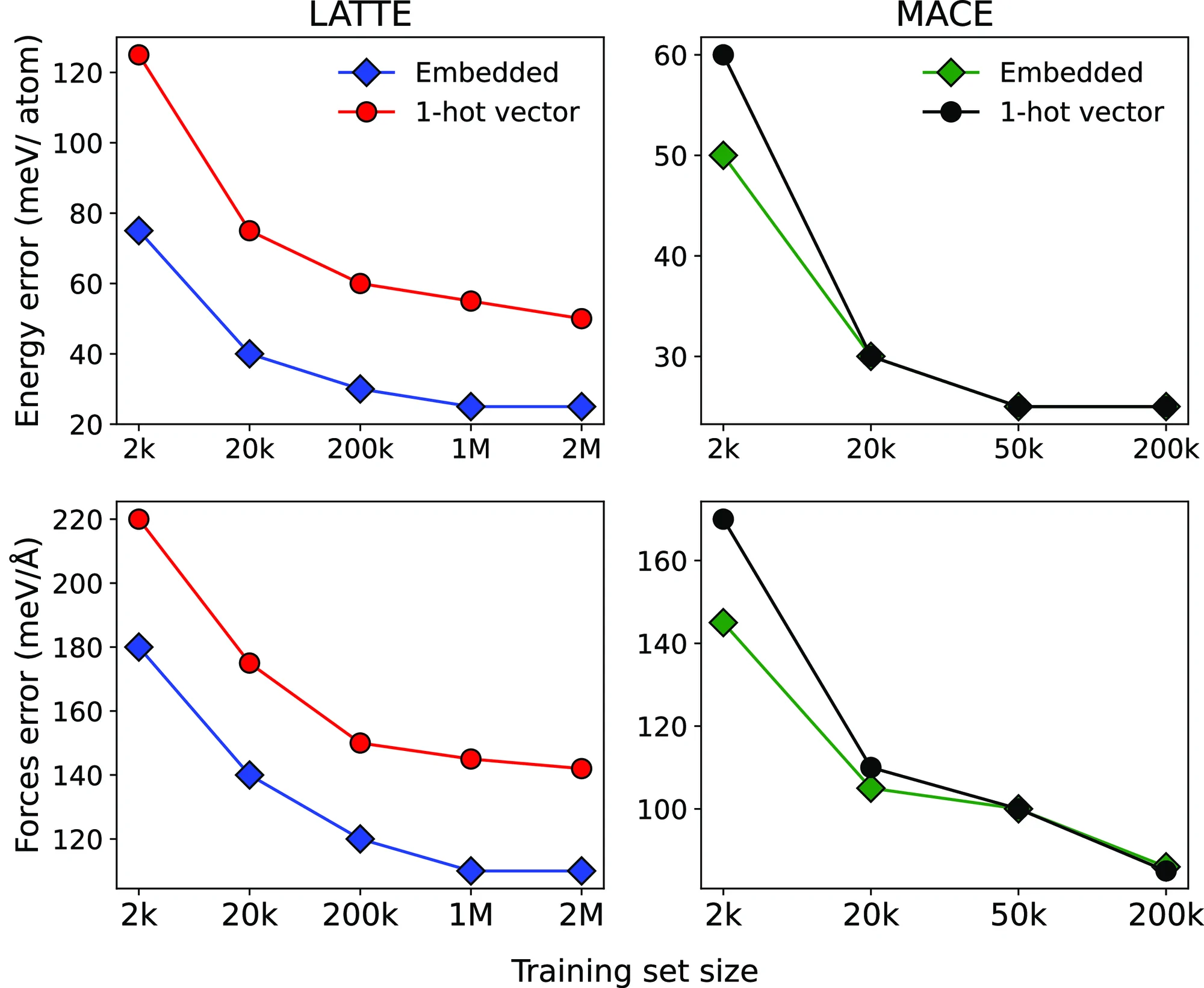
Data efficiency of LATTE (Left) and MACE (Right) models, trained on subsets of varying size, evaluated on a fixed subset of the OOD validation set.

To explore the architectural transferability of this finding, we investigated the possible impact of embedded atomic vectors in the MACE model. As shown in Figure 2 (bottom), atomic embeddings Matscholar significantly improved energy and force predictions in data-limited regimes, even for the already efficient MACE model. This advantage diminished with larger training sets, demonstrating that informative initialization matters most when data is scarce.

Meaningful atomic embeddings improve data efficiency for both descriptor-based (LATTE) and state-of-the-art graph-based (MACE) architectures. This shows that the benefits of learned representations are architecture-agnostic and provide a robust strategy for building accurate MLIPs when large training datasets are unavailable.

## 5 Conclusion and Outlook

The ability of machine-learned interatomic potentials (MLIPs) to handle diverse datasets and generalize beyond their training data remains a central challenge in computational materials discovery. In this work, we have demonstrated that two synergistic design principles—chemically informed atomic embeddings and cross-element parameter sharing—fundamentally enhance the transferability, efficiency, and scalability of MLIPs.

We showed that employing a meaningful feature vector as the elemental representation improves the prediction of the model for the same task. This feature vector, whether extracted from literature or elemental properties, introduces a logical direction to the model’s parameters, which are generated randomly. In our case study, we employ the feature vectors for the LATTE descriptor, and later we show that, even for a state-of-the-art model like MACE, having a logical elemental representation is a powerful approach to boost the model in low- data regimes. Moreover, employing the feature vector improves the robustness of the model’s generalization for out-of-domain structures and elements.

Through systematic evaluation across two distinct model architectures (LATTE and MACE) and the large, diverse OC20 dataset, we show that replacing simple 1-hot encoding with meaningful representations—such as property-based Magpie or literature-based Matscholar embeddings—reduces out-of-domain force errors by 20–30% and improves data efficiency by over 40% in low-data regimes. More strikingly, by strategically sharing parameters across elements, we reduce model sizes by up to 98% without loss in accuracy, and enable few-shot prediction for underrepresented elements with as little as 1% of the typical data.

These results collectively establish a practical and effective framework for building compact, generalizable, and data-efficient MLIPs. Our approach decouples model complexity from the number of chemical species and leverages pretrained embeddings to encode chemical intuition, directly addressing key bottlenecks in large-scale, multi-element materials exploration.

While our work shows that minimal data is still required for stable extrapolation to entirely unseen elements, the path toward true zero-shot capability is now clearer. Future research could explore dynamic or task-aware embeddings that adapt during training, or parameter-sharing schemes guided by learned elemental similarity. Applying this framework to more complex materials classes—such as high-entropy alloys, amorphous systems, or electrochemically dynamic interfaces—represents an exciting frontier.

The strategies presented here are architecture-agnostic and computationally lightweight. By integrating learned representations with shared parameterization, the next generation of MLIPs can be both more powerful and more practical—accelerating the discovery of new materials across the vast and uncharted regions of chemical space.

## Supplementary Material



## Data Availability

The training set (2M) and all of the 4 validation sets used in this work are available on the GitHub repository of the Open Catalyst Project https://opencatalystproject.org/. The code to reproduce results with the LATTE descriptor is publicly available in the PANNA package (https://gitlab.com/PANNAdevs/panna), at commit 132a2df662598ecbbb2c9b8d37b992bb6ada6348 (132a2df6).

## Notes

Details and hyperparameters of LATTE and MACE models, as well as modifications to the original MACE code https://github.com/ACEsuit/mace (PDF)
