# Supplementary material for: Enhanced Generalization through Atomic Embeddings and Cross-Element Parameterization
Source: J Chem Inf Model. 2026 Oct 2;66(20):13181–8. doi: 10.1021/acs.jcim.6c01725 (PMC13715492; doi:10.1021/acs.jcim.6c01725)
Supplement: Supplementary file 1 [file ci6c01725_si_001.pdf]

Supporting Information

Enhanced Generalization through Atomic Embeddings and Cross-Element Parameterization

Mina Taleblou,* Franco Pellegrini,* and Stefano de Gironcoli*

Scuola Internazionale Superiore di Studi Avanzati (SISSA), 34136 Trieste, Italy

E-mail: mtaleblo@sissa.it; pellefra@sissa.it; degironc@sissa.it

1 LATTE hyperparameters

A cutoff radius of 5 Å was used, with the descriptor containing 1000 terms: 100 2-body terms only sampling radial atomic distribution, 300 3-body terms of (α, α) type sampling vector-vector alignment, 300 terms of ($\alpha\beta$, $\alpha\beta$) type contracting 2nd order tensors, and 300 4-body terms of type (α, β, $\alpha\beta$) (shorthand notation: 100,-: 300,i,i: 300,ij,ij: 300,i,j,ij).

The LATTE descriptor was combined with a neural network with two hidden layers containing 240 and 192 nodes, respectively. A Gaussian activation function was applied to the input and hidden layers, while the last hidden layer was linearly connected to the output node. Training used the Adam optimizer[1] with a learning rate of 10^{-3} decaying to 10^{-4} during the first 100k steps, which remained constant thereafter.

The model was trained over batches of size 16, and the total loss was computed as the quadratic energy and force errors for the structures in each batch with equal weight for the energy and force contributions in the loss function. L1 regularization (with coefficient 0.001) was applied. A summary of the results of training LATTE with different sizes and types

of descriptors, different parameterizations, and embedding the central atom is presented in Table S1.

Table S1: Effect of number of terms in the descriptor (Descr) and number of nodes in each network layer (nodes) on prediction errors for energy (meV) and forces (meV/Å) for two types of parameterization.

d:$l_1l_2l_3$	Descriptor	nodes	ID		OOD-ads		OOD-cat		OOD-both	
			E	F	E	F	E	F	E	F
common-emb:css	100,-:300,i,i:300,ij,ij:300,i,j,ij	240:192:1	10	76	11	80	18	98	23	107
common-emb:css	20,-:20,i,i:30,ij,ij:30,i,j,ij	240:192:1	15	85	16	88	23	110	24	118
common-emb:css	100,-:100,i,i:100,ij,ij:100,i,j,ij	240:192:1	12	72	13	75	19	99	19	108
common-emb:css	150,-:350,i,i:350,ij,ij:350,i,j,ij:350,i,jk,ijk	240:192:1	10	74	11	77	18	96	18	104
common-emb-central:ccc	100,-:300,i,i:300,ij,ij:300,i,j,ij	240:192:1	11	70	12	73	19	102	19	110
common-emb-central:ccc	100,-:300,i,i:300,ij,ij:300,i,j,ij	1024:512:256:1	12	71	13	73	19	103	19	120

In Table S1, the weight of force loss in the loss function was set to be half of the one associated with the energy.

2 MACE hyperparameters and embedding implementation

A model with 64 channels in the atomic attribute description was trained on the OC20 dataset. Direct atomic interactions were calculated within a cutoff radius of 6 Å up to 4-body at each layer, and further atoms communicate through 2 message-passing layers. The default value of 1 was assigned to the symmetry of the messages for equivariant information exchange between neighborhoods.

The inclusion of embedding matrix information in the initialization of the elemental attribute array is implemented as follow in the MACE source:

```
class MACE(torch.nn.Module):
    def __init__():
```

```
.......

 % Embedding
node_attr_irreps = o3.Irreps([(num_elements, (0, 1))])
node_feats_irreps = o3.Irreps([(hidden_irreps.count(o3.Irrep(0, 1)), (0, 1))])

if isinstance(embedding_mat, np.ndarray):
self.embmat = True
self.full_emb_dim = full_emb_dim
print('full_emb_dim', full_emb_dim , flush = True)
self.register_buffer(
"embedding_mat", torch.tensor(embedding_mat, dtype=torch.get_default_dtype())
)
self.emb_size = embedding_mat.shape[1]
self.node_embedding = LinearNodeEmbeddingBlock(
irreps_in=o3.Irreps(f'{self.emb_size}x0e'), irreps_out=node_feats_irreps * Emb vector in
)
print('node_embedding: ', self.node_embedding, flush = True)
else:
self.embmat = False
self.full_emb_dim = 0
self.embedding_mat = None
self.emb_size = 0
self.node_embedding = LinearNodeEmbeddingBlock(
irreps_in=node_attr_irreps, irreps_out=node_feats_irreps
)
.....
```

```
def forward(...):
.....

 % Embeddings
if self.embmat:
node_feats = self.node_embedding(
torch.matmul(data["node_attrs"],self.embedding_mat))
if self.full_emb_dim>0:
node_attrs = torch.matmul(data["node_attrs"],self.embedding_mat)
else:
node_attrs = data["node_attrs"]
else:
node_feats = self.node_embedding(data["node_attrs"])
node_attrs = data["node_attrs"]
vectors, lengths = get_edge_vectors_and_lengths(
positions=data["positions"],
edge_index=data["edge_index"],
shifts=data["shifts"],
)
```

References

(1) Kingma, D. P.; Ba, J. Adam: A Method for Stochastic Optimization. 2017; https://arxiv.org/abs/1412.6980.
